# Depression and Coronary Heart Disease

**DOI:** 10.5402/2012/743813

**Published:** 2012-11-22

**Authors:** Karina W. Davidson

**Affiliations:** Center for Behavioral Cardiovascular Health, Department of Medicine, Columbia University, New York, NY 10032, USA

## Abstract

There are exciting findings in the field of depression and coronary heart disease. Whether diagnosed or simply self-reported, depression continues to mark very high risk for a recurrent acute coronary syndrome or for death in patients with coronary heart disease. Many intriguing mechanisms have been posited to be implicated in the association between depression and heart disease, and randomized controlled trials of depression treatment are beginning to delineate the types of depression management strategies that may benefit the many coronary heart disease patients with depression.

## 1. Introduction

Depression is quickly becoming the leading cause of years of life lived with disability worldwide [1] and has a particularly large impact on compromised health when comorbid with a chronic medical disorder [2]. More than 17 million American adults survived after an acute coronary syndrome (ACS), with 1.2 million new surviving cases added per year [3]. More than 2 out of every 5 of these patients have significant patient-reported depressive symptoms [4], and these symptoms will remain long after discharge [5]. Thus, as many as 7 million of Americans living with coronary heart disease (CHD) are also suffering with clinically significant depression, and we add half of a million new cases to this public health burden annually. As the comorbidity between these two conditions is high, and both predict increased occurrence of the other, a whole field has emerged to study the complex inter-relations of depression and ACS. For example, presence of a depressive disorder and/or minimally elevated depressive symptoms appears to be a robust risk and prognostic marker of CHD recurrence and all-cause mortality [4, 6, 7]. In this paper, the definition, measurement, epidemiology, mechanisms, screening, and treatment recommendations for depression comorbid with coronary heart disease will be presented, as will the controversies and future research directions in this interesting intersection between mental and physical disease. 

## 2. Defining Depression

Many researchers and much of the lay public assume that depression is a categorical disorder, and that those who suffer with symptoms typically associated with depression have a disease qualitatively different from everyday distress. Consequently, they consider that this cluster of symptoms should be viewed as one of a series of psychiatric illnesses, as described within the Diagnostic and Statistical Manual of Mental Disorders (DSM) [8]. However, there are others promoting continuous or dimensional views of these symptoms. Both conceptualizations are reviewed, starting with disease categories because some of the debates within psychiatric nosology may shed light on the depression—CHD association.

### 2.1. Depression as a Psychiatric Disorder

Depressive disorders are syndromal diagnoses based on subjective experience of psychological and somatic symptoms that are reported to a professional who evaluates these symptoms along continua of intensity, duration, and impact on daily functioning. Categories from the DSM-IV [8] include major depressive disorder, dysthymia, adjustment disorder with depressed mood, depression due to a general medical condition, and depression not otherwise specified (n.o.s.). 

Differential depression disorder diagnosis is decided based on the duration of the illness, the number of symptoms present, and the presumed etiology of the illness—all of which are distinctions that should be reported when investigating the link between depression and CHD. *Major Depressive Disorder* (MDD) is the most commonly studied clinical diagnosis in relation to CHD. It is characterized by clinically significant impairment in at least five areas such as appetite or sleep that have persisted for at least two weeks, plus the presence of depressed mood or the loss of pleasure/interest for most of the day during those two weeks. In contrast, *dysthymia* is reserved for a cluster of symptoms that persisted over a longer period. The number of symptoms needed to diagnose dysthymia is less than in MDD, but persistent depressed mood has to be present throughout two years. Both MDD and dysthymia can be further specified according to the presence or absence of atypical symptoms such as reactive mood, hyperphagia (i.e., excessive eating), hypersomnia, leaden paralysis, or longstanding interpersonal rejection sensitivity. 

The diagnosis of *mood disorder due to a general medical condition* describes a significant disturbance in mood that is directly related to the presence of a medical condition known to cause depression, as long as the mood symptoms do not meet criteria for other DSM-IV categories of depression. Thus, there must be evidence that the psychiatric symptoms are actually caused by the medical condition rather than being just a reaction to being ill. One of the most clearly documented examples of this type of depressive disorder is the depression that results from Parkinson's disease [9]. Although there is a high prevalence of elevated depressive symptoms after an acute coronary syndrome (ACS) event, no one has yet proposed that the ACS may cause these symptoms; however, depressive symptoms can result from cerebrovascular disease [10]. Thus, one intriguing possibility is that depression due to a medical condition (vascular depression in in this case) may be precipitated by the same CVD risk factors as those responsible for the ACS. 

Next, *adjustment disorder* is characterized by the development of clinically significant emotional or behavioral symptoms in response to an identifiable psychological stressor. The symptoms must develop within three months after the onset of the stressor and must resolve within six months of the termination of the stressor [11]. Severe physical events, such as an ACS, can be a precipitant of an adjustment disorder with depressed mood, but extreme mood disturbance would still be classified as a major depressive disorder in DSM-IV, and symptoms lasting longer than six months would similarly be classified as meeting criteria for a mental disease. Might it be adjustment disorder that predicts ACS recurrence? Only well-trained diagnostic staff, alert to the sometimes subtle distinction between adjustment and other depressive disorders, could address this question, and no such study has classified sufficient numbers of ACS patients with adjustment disorder to test this intriguing hypothesis. 

*Depression n.o.s.* allows the classification of less severe syndromes of depression (with fewer symptoms) of a shorter duration (less than 2 weeks) that do not meet criteria for major depressive disorder or dysthymia. It encompasses DSM-IV research categories such as minor depression and brief recurrent depressive disorder, which have also been labelled as “*subthreshold*” *depression*, a category that encompasses syndromes of depression that fail to meet criteria for major depressive disorder [12], but which show some significant impairment in social or occupational functioning [13]. “Subthreshold” depression has been proposed by investigators conducting longitudinal studies of mood disorders (e.g., [14, 15]) to reflect normal fluctuations in the longitudinal course of mood disorders, and a similar construct has been found to predict CHD recurrence [16, 17]. Thus, the longitudinal course of depressive symptoms, as well as subthreshold depression levels, must be carefully assessed to determine if one or both are critical to ACS prognosis. 

More recent approaches to categorical classification have addressed the problem of comorbid anxiety and depressive illnesses. For example, a study by Kessler et al. [18] found that up to 58% of patients with a lifetime diagnosis of major depressive disorder may suffer from anxiety disorder(s), suggesting that the psychiatric diseases' underlying pathophysiologies may not respect DSM-IV boundaries. The broad implications of this highly prevalent comorbidity are that etiologies [19], neuroendocrine mechanisms [20], and sequelae may be common to both diseases, so ACS patients should be assessed for both anxiety and depressive disorders, as well as histories of both, to more fully understand the association of depression to ACS recurrence. 

### 2.2. Assessment of Depression as a Psychiatric Disorder

Common assessment tools for reliable diagnosis of depressive disorders include the Structured Clinical Interview for DSM-IV (SCID; [21]) and the Diagnostic Interview Schedule (DIS; [22]). The SCID is a semistructured interview designed for administration by a clinician or trained mental health professional to provide a basis for accurate DSM-IV diagnoses. It exists in several versions (e.g., SCID-I for DSM Axis I diagnoses, SCID-II for DSM Axis II (personality disorders) diagnoses), and every diagnostic category (e.g., mood disorders) has its own module that can be used separately. A research version is available which can be modified, and research assistants without a clinical degree can reliably administer this version after extensive training. Test-retest reliability of the SCID ranges widely according to setting and study population (e.g., Williams et al. [23]). Its validity has not been extensively studied mostly because of the lack of a “gold standard” for psychiatric diagnoses. In contrast, the SCID itself is widely used as the gold standard against which the sensitivity and specificity of self-report measures is evaluated.

The *DIS* can also be administered by trained nonclinician interviewers. Its answers are completely precoded which enhances reliability; however, the DIS is rather inflexible and some have argued that it is insensitive to change [24]. The DIS served as one basis for the development of the Depression Interview and Structured Hamilton [24] (DISH: a semistructured interview developed for the Enhancing Recovery in Coronary Heart Disease (ENRICHD) study). 

The *DISH* was designed to assess the severity of depressive symptoms and enable trained interviewers to make a DSM-IV major depression, minor depression, and dysthymia diagnosis in medically ill patients. Furthermore, the lifetime and family history of these diagnoses is assessed. The DISH was validated against the SCID (88% diagnostic overlap) and in ENRICHD, clinicians agreed with 93% of diagnoses made by research nurses [24]. It is likely the best depression interview to be used with ACS patients, to accurately determine if depression exists, rather than somatic symptoms resulting from the cardiac disease. 

### 2.3. Depression as Self-Reported Distress Symptoms

When professionally trained interviewers are not available and/or it is not feasible to conduct psychiatric diagnostic interviews, reliance on self-report measures of depression for diagnostic classification has been common in studies of CHD patients [25]. There are many such measures, each with its own merits, and some available in updated and/or shortened versions. Each of these self-report measures was designed to detect depressive disorders in various populations (such as primary care patients, or older adults, or population-based samples) and they detect depressive disorders and demonstrate item consistency, split-half reliability, and stability to varying, but generally satisfactory degrees [25, 26]. Two of the most frequently used, the Beck Depression Inventory (a trademarked inventory—both versions I and II (see http://www.pearsonassessments.com/ for ordering instructions)) [27, 28] and the Center for Epidemiological Studies—Depression subscale (CES-D [29]), are also frequently employed in studies of depressive symptoms and CHD, and both have shown good predictive validity to ACS events [30, 31]. However, of concern in choosing any of these measures for use in a depression—CHD study is their sensitivity and specificity for depression detection in older patients with comorbid medical illnesses, who have recently undergone a life-threatening medical crisis [32]. 

Unfortunately, discrepancies between what investigators mean by the term “depression,” and how they then assess it, may lead to erroneous conclusions, contradictory findings, and difficulty in summarizing the evidence-base of the inter-relationships between depression and CHD that has—or has not—accrued.

## 3. Epidemiology and Prognostic Significance of Depression Comorbid with Coronary Heart Disease

### 3.1. Depression in CHD Patients Is Prevalent

An Agency for Healthcare Research & Quality systematic review reported that up to 20% of CHD patients meet criteria for a major depressive disorder, and up to 47% have significant patient-reported depressive symptoms, and these diagnoses/symptoms remain long after discharge [5]. Thus, as noted in a comprehensive review by Carney, almost 2 out of every 5 ACS patients have clinically significant depression [4]. This contrasts starkly with the general population, where depression is seen in 4 to 7% persons [33]. When DSM criteria are used to establish a diagnosis, prevalence tends to be somewhat lower (i.e., 15 to 20%) in CHD patients, but even minor depression or elevated depressive symptoms are seen in 30 to 50% of these patients [34]. Importantly, both clinically diagnosed depression and elevated depressive symptoms predict increased cardiac risk, as described below.

### 3.2. Depression in CHD Patients Is Observationally Associated with Diminished Health-Related Quality of Life

Depression in both stable CHD patients and those with a recent ACS clearly predicts impoverished health-related quality of life, independent of traditional predictors of quality of life [35]. For example, recent ACS patients with a history of depression have twice the rate of reported angina, triple the reported physical limitations, and are at almost triple the risk of diminished health-related quality of life [36]. Of multiple predictors of one-year quality of life in a study of myocardial infarction (MI) patients, including demographic and social variables, severity of disease, and other predictors, depression was the most important [37]. In another large study of CHD patients, depression was once again the most important correlate of diminished quality of life, while 2 traditional measures of cardiac function, ejection fraction and ischemia, were not [38]. In fact, a recent review demonstrated that depression was more highly associated with health-related quality of life & health status than angina, arthritis, asthma, or diabetes [39]. There have been calls to improve quality of life in post-ACS patients, rather than continue to focus on extending life of diminished quality [40]. Treating depression could answer this call. 

### 3.3. Depression in CHD Patients Is Observationally Associated with High Costs

Depression has long been associated with high costs of medical utilization [42], many lost days of productivity, and reduced performance while at work [43]. Patients who have a chronic medical condition such as CHD, and comorbid depression, have significantly more ambulatory visits, emergency room visits, days in bed due to illness, and functional disability [44]. Five-year cardiovascular health costs were 15–53% higher in women with myocardial ischemia who were depressed compared to those who were not depressed [45]. In post-MI patients with elevated depressive symptoms, one year health care costs of out-patient, emergency room, and readmission visit costs (*excluding* mental health costs) were 41% higher than for post-MI patients with few depressive symptoms [46].

### 3.4. Depression in CHD Patients Is Observationally Associated with Poor Medical Prognosis

**Figure 1:**
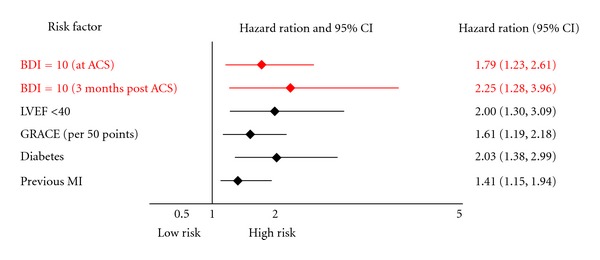
Hazard ratios of depressive symptoms and traditional cardiovascular risk factors.

In addition to increased health care utilization, death and morbidity are societal costs associated with depression in post-ACS patients. Large epidemiological studies have demonstrated convincingly that depression is a predictor for occurrence and recurrence of CHD. Depressive symptoms alone also predict CHD risk, but stronger effect sizes have been observed for MDD compared to depressive mood, suggesting a dose-response relationship [31, 47, 48]. In addition to the enormous patient and caregiver burden, post-MI patients who are depressed have more medical comorbidities [49] and cardiac complications [50] and are at greater risk for mortality compared to nondepressed post-MI patients [16, 49–51]. Prospective observational studies show, among ACS patients, the hazard ratio associated with depressive symptoms is 1.80 (95% CI: 1.46–2.51) for all-cause mortality [52], and 1.95 (95% CI: 1.33–2.85) for MI recurrence [6]. A recent meta-analysis indicates that elevated depressive symptoms continue to strongly identify CHD patients at mortality risk (relative risk: 3.41 (95% CI: 2.19–5.31)) [52]. As can be seen in Figure 1, depressive symptoms in CHD patients is on par with more conventional CHD recurrence prognostic factors for predicting death and CHD recurrence.

The biology of depression suggests dysregulation of several physiological systems, some of which are implicated in the depression-CHD recurrence link [53]. However, the evidence remains equivocal regarding which biological dysregulations are responsible for the link between depression and CHD.

## 4. Mechanisms by Which Depression May Confer Coronary Heart Disease Recurrence and Death Risk

There are many behavioural and biological mechanisms that have been proposed to explain the association between depression and CHD [53, 54]. Biological mechanisms proposed to explain the depression-CHD prognosis association include platelet reactivity, inflammation, autonomic imbalance, sleep architecture disruption, circadian rhythm disruption anabolic/catabolic hormonal imbalance, as well as many others [55–58]. A selected review of the evidence available for many of the proposed mechanisms is provided below. 

### 4.1. Evidence for the Link between Platelet Reactivity and Depression

Several case-control studies have demonstrated the existence of platelet hyperreactivity in CHD patients [59, 60]. In a study by Laghrissi-Thode et al. [60], higher levels of platelet factor 4 and *β*-thromboglobulin, markers of platelet aggregation, were described in MDD patients with CHD compared with nondepressed CHD patients. Serebruany et al. [61] pooled the data from 3 studies of post-ACS patients: ASSENT-2, PRONTO, and SADHART, the last of which specifically screened and enrolled MDD patients. Fifty nonsmoking, nondiabetic subjects without cardiovascular disease were also enrolled as controls. The study showed that patients with CHD and MDD exhibited the highest levels of platelet factor 4, *β*-thromboglobulin, and platelet/endothelial cell adhesion molecule-1 when compared with MI or unstable angina patients without MDD.

### 4.2. Evidence for the Link between Inflammation and Depression

Several cross-sectional studies have linked depression to chronic inflammation in otherwise healthy participants [62–64], as well as in post-ACS patients shortly after the index event [65, 66]. Elevated levels of inflammatory biomarkers including c-reactive protein (CRP), soluble intercellular adhesion molecule 1 (sICAM1), soluble vascular cell adhesion molecule-1, and tumor necrosis factor-*α* (TNF-*α*) are associated with an increased risk of cardiovascular events in patients with known CHD including survivors of a recent ACS [67–70].

CRP is significantly higher in depressed patients with no CHD compared with matched nondepressed controls. The cardiovascular health study enrolled patients aged 65+ years with no CHD history and found that participants with increased depressive symptoms had higher CRP levels, after adjusting for potential confounders [71]. Similarly, recent findings from the ATTICA study demonstrated that depressive symptoms were positively correlated with higher CRP in 853 participants [72]. A recent analysis of the National Health & Nutrition Examination (NHANES III) survey found that patients with a history of MDD had elevated CRP levels compared to patients with no history of depression (OR = 1.64), independent of other standard CHD risk factors [73]. Data also show that depressed patients have elevated peripheral white blood cell counts and increased B- and T-cell activity [74–77]. 

Similarly, sICAM1 has been cross-sectionally associated with depression [78]. A recent study showed that the expression of sICAM-1 on vascular endothelial cells in the brain was associated with depression development [79]. In addition, psychological stress (often associated with depressive symptoms) increases sICAM-1 expression in circulating granulocytes, lymphocytes, and monocytes [78].

Findings are similar for post-ACS patients. Four recent studies have examined the relation between depression and inflammatory biomarker levels after an ACS. Lespérance et al. [80] showed that levels of sICAM-1 was related to depression at a mean followup of 2 months after the ACS. Additional analyses revealed that a significant relationship was found between depression and elevated CRP levels only in patients not taking statins. Miller et al. [81] showed that depression was associated with increased CRP levels independent of several possible confounders including statin therapy in patients who had an acute coronary syndrome event or coronary revascularization ≥3 months prior to enrollment. There was no association between depression and levels of interleukin-6 (IL-6) or TNF-*α*. Similarly, we found that persistently elevated levels of depression 3 months following an acute coronary syndrome event were significantly associated with elevated CRP levels, independent of several possible confounders [82]. This was the case in both patients who were prescribed statins at any time during the three month period, and in those who were not. In contrast, only one study has shown no significant association between depression and elevated inflammatory biomarkers. Annique et al. [83] showed that there was no relation between several inflammatory markers such as CRP, TNF-*α*, IL-6, and neopterine in depressed versus nondepressed patients post-ACS at a mean followup of 6 months. 

The immune system usually reacts to an inflammatory stimulus with an induction of a T helper 2/anti-inflammatory shift to protect the organism from excessive T helper 1/proinflammatory cytokine release. However, stress hormones such as cortisol are capable of facilitating inflammation through further inducing the release of proinflammatory cytokines without the adaptive simultaneous release of anti-inflammatory cytokines. In addition to an elevation in proinflammatory markers, a reduction in anti-inflammatory markers may also be associated with depression. Interleukin-10, an anti-inflammatory cytokine, was examined in chronic heart failure patients with and without depressive symptoms. Those with depressive symptoms exhibited significantly lower levels of interleukin-10 and higher levels of the proinflammatory cytokines TNF-*α* and IL-6 [84]. These findings suggest that depression is associated with a downregulation of anti-inflammatory cytokines with a simultaneous upregulation of proinflammatory cytokines.

In summary, several studies indicate that depression in CHD patients is associated with an increase in proinflammatory and a decrease in anti-inflammatory cytokines, which are both associated with an increased risk of recurrent cardiovascular events. Thus, a dysregulation in the inflammatory pathways may mediate the link between depression and recurrent CHD.

### 4.3. Evidence for the Link between Autonomic Dysregulation and Depression

Autonomic dysregulation is characterized by increased activation of the sympathetic nervous system (SNS), which usually acts in concert with a reduced activation of the parasympathetic nervous system (PNS). Both elevated SNS activity and reduced PNS activity have been implicated in depression and CHD recurrence. 

Several studies have documented that depressed individuals compared to their nondepressed counterparts have lower heart rate variability (HRV), a well-established noninvasive index of cardiac autonomic regulation, although these differences may be seen in different frequency bands. Using data from the ENRICHD trial, Carney and colleagues demonstrated that very low frequency (VLF) HRV was lower in depressed post-MI patients compared to their nondepressed counterparts and partially mediated the relationship between depression and mortality after MI [85]. While the underlying physiology of VLF HRV is not well understood, the fact that it, along with low frequency (LF) and high frequency (HF) power, is virtually eliminated by the parasympathetic antagonist atropine suggests a substantial vagal contribution [86]. In another analysis from the ENRICHD dataset, HF, LF, VLF, and ultra-low frequency (ULF) HRV were all lower in depressed versus nondepressed post-MI patients [65]. After adjustment for covariates, these differences remained statistically significant for all but HF power. In healthy women from the Women's Health Initiative, several time domain indices of HRV were lower among depressed participants compared to those with no evidence of depression [87]. While there are exceptions [88, 89], these data suggest that depression is associated with reduced cardiac parasympathetic modulation.

Research on the relation between the activity of the SNS and depression has a long history. Several studies have shown increased catecholamine levels in depression, although there have been exceptions [90, 91]. Esler et al. [92] found increased norepinephrine (NE) spillover. The rate of norepinephrine released to plasma from sympathetic nerves in some but not all depressed patients. Wong and colleagues measured cerebrospinal fluid (CSF) NE and plasma cortisol each hour for 30 consecutive hours in controls and patients with melancholic depression (MDD with specific additional features including anhedonia or loss of interest and pleasure in things or people) and demonstrated higher CSF NE and plasma cortisol levels around the clock in the depressed patients [93]. Veith and colleagues showed that depressed patients had an increased rate of NE entry into the circulation [94]. Other studies also have shown elevated levels of plasma NE in depressed patients [95–97]. Recently, Gold et al. demonstrated that severely depressed patients had increased heart rate (HR), blood pressure (BP), CSF NE, and plasma NE compared to a control group [98]. In a recent study of 91 women, higher levels of depression were positively associated with 24-hour urinary NE excretion [99]. The same pattern of results emerged from the Heart and Soul Study [100]. Among 598 patients with coronary heart disease, those with depressive symptoms (*n* = 106) had greater mean 24-hour urinary norepinephrine excretion levels and were more likely to have norepinephrine excretion levels in the highest quartile and above the normal range than those without depressive symptoms. Thus, there is substantial evidence that both serum and urinary norepinephrine are typically elevated in depressed patients.

### 4.4. Evidence for the Link between Autonomic Dysregulation and CHD

There is abundant evidence that the autonomic nervous system plays a significant role in the pathophysiology of CHD recurrence. By enhancing myocardial electrical stability, the parasympathetic nervous system reduces the risk of sudden cardiac death. Animal experiments confirm the capacity of higher levels of cardiac vagal regulation to protect against sudden death after experimentally induced MI [101], and HRV is a powerful predictor of sudden death in heart failure patients [102] and in patients at elevated risk for sudden death [103]. This effect may account for the repeated finding that low levels of HRV confer elevated risk of adverse events following an MI [104–106]. In post-CABG patients randomized to the placebo condition in a trial of gemfibrozil, low levels of HRV predicted the progression of coronary atherosclerosis after controlling for conventional risk factors [107].

Excess activity of the sympathetic nervous system produces many effects that contribute to CHD recurrence in post-ACS patients. It raises blood pressure, increases myocardial oxygen demand, activates platelets, induces myocyte apoptosis, and is arrhythmogenic. Administration of *β*-blockers, which protect against future events in post-MI patients, is evidence of the role of SNS in adverse events. Correspondingly, research suggests that diurnal changes in indices of SNS activity, for example, catecholamines, are associated with the diurnal pattern of the onset of cardiovascular events, which more frequently happen in the morning hours [108–112], with a meta-analysis concluding that 1 in 11 myocardial infarctions are attributable to the morning excess incidence [113]. Systemic a2-receptor blockade by yohimbine reduced the sympathetically-mediated orthostatic increase in platelet aggregation significantly, further suggesting the central role of the SNS in the risk of thrombotic vascular events in atherosclerosis [114]. Patients with evidence of coronary endothelial dysfunction show increased vasoconstriction in response to catecholamines [115].

### 4.5. Evidence for the Link between Sleep Architecture Disruption and Depression

Depression and sleep architecture disruption are closely linked, although identification of the specific polysomnographic parameters that are uniquely dysregulated in depression remains controversial [116].

Total sleep time is decreased in depressed patients and those prone to depressive episodes [117], and while prospective data are lacking, disturbances that shorten sleep duration (prolonged sleep latency, early morning awakening, sleep continuity disturbances, and insomnia) have all been suggested as possible contributors to depressed mood [118]. Cross-sectional studies of depressed versus nondepressed patients regularly find that total sleep time is almost always reduced in the depressed patients [116, 117, 119].

Reduced REM latency, the time from sleep onset to the first occurrence of REM is the most frequently reported sleep dysregulation that distinguishes MDD patients from normals, other psychiatric patients, and those with nonendogenous depression Benca 1992 [120]. A meta-analysis drew the same conclusion [116].

Depressed patients tend to have decreased slow wave sleep as reviewed by a meta-analysis incorporating results from 177 studies with data from 7151 patients and controls [116, 119, 121]. More recently, Armitage et al. reported that compared to healthy subjects, slow wave sleep was decreased in depressed outpatients [122]. Furthermore, antidepressant use appears to reverse these sleep markers of depression, by increasing total slow wave sleep and the slow wave sleep in the first sleep cycle, but not in the second cycle [123]. 

### 4.6. Evidence for the Link between Sleep Architecture Disruption and CHD

While prospective epidemiological studies relating many of the dimensions of sleep architecture to CVD recurrence are lacking, there is epidemiological evidence that short sleep duration is predictive of ACS incidence [101, 124]. REM sleep is characterized by pronounced surges of sympathetic activity [125] which may be of sufficient magnitude (a) to stimulate thrombotic processes, (b) to increase hemodynamic stress on vessel walls conducive to plaque rupture, and (c) to alter cardiac electrophysiological properties [126]. These autonomic surges could be responsible for cardiac events witnessed during REM sleep in humans [127, 128]. Importantly, this REM-induced cardiac sympathetic dominance is even more enhanced in individuals with recent MI [129]. There is no evidence addressing the possible role of slow wave sleep in predicting either ACS occurrence or recurrence, but lack of restorative vagal regulation could underly the association with ACS recurrence. Thus, we only have indirect evidence that sleep architecture may be implicated in CHD RECURRENCE/DEATH, but we do have theoretical foundations for positing this association. 

### 4.7. Evidence for the Link between Circadian Rhythm Disruption and Depression

Endogenous circadian rhythms regulate daily variations in most of the hormonal, physiological, and psychological variables implicated in depression as well as ACS [130]. The systems with the most prominent variations are thermoregulation or core temperature, and melatonin.

Core temperature, defined as the operating temperature of an organism, specifically in deep structures of the body, is seen as the gold standard for measurement of circadian rhythm. Elevated nocturnal body temperature is one of the more consistently observed circadian abnormalities in depression [131–136]. Several studies show that during the depressed phase, average temperature rises and temperature amplitude (maximum temperature minus minimum temperature) decreases. Effects of antidepressants on the core body temperature of depressed patients include (a) lowering of the minimum temperature during the night, and (b) increasing temperature amplitude [133]. Electroconvulsive therapy increases circadian amplitude and lowers core body temperature in depressed subjects [136].

Melatonin is a pineal indoleamine derived from serotonin and is a reliable marker of circadian rhythmicity in humans. Melatonin concentrations are increased at night and are undetectable during the day. Depression-related disturbances in the melatonin circadian profile in the form of advanced phases and/or decrease in nocturnal amplitude were demonstrated as early as 1979 [137–139] and have been subsequently replicated [140–149].

### 4.8. Evidence for the Link between Circadian Rhythm Disruption and CHD

There is evidence that the majority of cardiovascular events (including myocardial infarctions) show a marked circadian rhythmicity with a peak incidence between 6 am and 2 pm [113, 150]. However, in depressed patients, for whom there are often circadian dysregulations, the most prevalent time for myocardial infarctions is between 10 pm and 6 am [151]. Thus, circadian rhythm disruption in depressed patients may help elucidate some of the pathways by which these patients are at increased risk for recurrent cardiovascular events.

### 4.9. Evidence for the Link between Anabolic/Catabolic Hormone Imbalance and Depression

An adequate balance between catabolic processes (energy mobilization), which are induced by stress hormones such as catecholamines and cortisol, and anabolic processes (repair, healing, growth), which are induced by sex steroids, among others, is essential for health and survival. Dehydroepiandrosterone (DHEA), an anabolic sex steroid precursor, a marker frequently used in combination with cortisol to capture anabolic/catabolic hormone imbalance, has been implicated in protection against coronary artery disease [152–154].

The hypothalamic-pituitary-adrenal (HPA) axis, the major stress axis through which cortisol is released by the adrenal gland when stimulated by adrenocorticotrophin (ACTH), has been studied extensively in depression. Depressed patients exhibit elevated circulating plasma levels of ACTH and cortisol, and elevated urinary cortisol concentration [155]. 24-hour sampling studies have found increased cortisol secretion in depressed persons [155–158], as well as alterations in the circadian rhythm of cortisol [155–158]. Most studies on morning salivary cortisol have demonstrated an increased awakening response in depressed versus nondepressed subjects [159–161] but not all have found this [162]. In 781 subjects from the CARDIA study of heart disease in young adults, depression was associated with flatter diurnal cortisol rhythm [163]. Pharmacological challenge studies indicate diminished negative feedback in the HPA axis in depressed persons [164].

There are several studies on the association of anabolic/catabolic hormone balance with depression. The cortisol/DHEA ratio was greater in elderly patients with MDD compared with age-matched healthy controls [165]. In adolescents, high morning cortisol/DHEA ratios predicted persistent MDD [73]. In a nonclinical sample of older men, a high morning cortisol/DHEA ratio was associated with high anxiety, negative mood, and deficits in various aspects of cognitive function [166]. Young and colleagues showed that a high cortisol/DHEA ratio in the evening was associated with depression in an adult population [167]. In another study, patients with major depressive disorder comorbid with borderline personality disorder displayed elevated cortisol/DHEA ratios relative to normal controls [168]. In sum, there is evidence that anabolic/catabolic hormone imbalance is observed in depression. Variation in study results is likely to be due to the heterogeneity of depression as a phenotype and the lack of standard measurement of this phenotype. 

### 4.10. Evidence for the Link between Anabolic/Catabolic Hormone Imbalance and CHD

Bain and colleagues prospectively investigated serum cortisol during hospitalization for acute MI and found that very high levels of cortisol (>2000 nmol/L) predicted mortality [169], a finding that was replicated by Karga and colleagues [170]. A high cortisol/DHEA ratio is thought to create an imbalance between protein synthesis and degradation favoring catabolism over anabolism in patients with chronic heart failure [171]. We know of no studies demonstrating that an imbalance is associated with CHD recurrence/death outcomes in post-ACS patients. Cortisol and DHEA are both secreted in response to ACTH released from the pituitary gland, hence, the two steroids tend to correlate. In a study in intensive care unit patients, age-adjusted correlations between early morning DHEA and cortisol ranged between 0.47 (surgical intensive care unit patients), 0.62 (medical intensive care unit patients), and 0.69 (neurologic intensive care unit patients) [172]. In contrast to cortisol, DHEA has been associated with antidepressive and neuroprotective properties, and the cortisol/DHEA ratio has been discussed as a measure of the relative activity of the two steroids with two types of hormone profiles posing an “endocrine risk”: higher cortisol levels with normal DHEA, and normal cortisol levels with lower DHEA leading to the same functional outcome [173]. 

### 4.11. Summary of Biological Mechanisms

While many promising mechanisms have been reviewed, we have little direct human evidence that any of these are causally implicated in the pathogenesis associated with depression comorbid with CHD. A recent review of animal studies [174] suggests that most of the above mechanisms continue to be plausible, but until we move into human experiments or trials, there remains many future studies that are required to definitively answer which biological mechanisms are implicated in the depression—ACS recurrence association. 

### 4.12. Evidence for the Link between Behavioral Mechanisms and Depression

Depression may also influence post-ACS outcomes through its effects on a patient's *behaviors*, such as adherence to prescribed medications [175, 176] and/or to secondary prevention recommendations after ACS [177]. In addition, there may be disparities in the way the healthcare system behaves towards depressed patients, and these differences, for example, the treatment they receive, may lead to worse outcomes [178]. Although it is now widely accepted that depression after ACS predicts poorer medical prognosis, there remains a gap in our knowledge about which of the proposed behavioral mechanisms might explain this association. 

Prior research shows that overall, a diagnosis of depression is associated with poor adherence among patients with a number of chronic medical illnesses, including HIV [179], hypertension [180], diabetes [181], and ACS [182]. Our own research has shown that patients with persistent depressive symptoms after ACS are also less likely to adhere to secondary CHD prevention behaviors such as exercising regularly and quitting smoking [177]. Furthermore, we have data showing that as compared to nondepressed patients, patients with depression are at greatest risk for poor medication adherence [183]. 

In the case of ACS, not only secondary prevention behaviors, but behaviors pertaining to managing ACS symptoms have been shown to impact medical outcomes. For example, delay in the time from when patients develop symptoms of chest pain to the time that they present for medical care is clearly associated with worse post-ACS prognosis [184]. A recent publication indicates that depressed patients take on average 12 hours longer than nondepressed patients to present to ER [185]. While intermediary depression phenotypes were not differentiated in this sample, it may be the Melancholic depressed patients who largely explain this difference.

Finally, as a result of the cognitive, affective, and social characteristics of mental illnesses, patients with depression can be stigmatized by their illness [186]. This stigma may lead to lower rates of treatment for cardiac disease, or poorer communication about secondary prevention behaviors. Patients with depression tend to have flat affect and to be less engaging; they therefore may be *most susceptible* to such physician bias. Indeed, differences in the receipt of ACS treatment among patients with and without mental illness have been documented. Druss et al. showed that individuals with co-morbid mental disorders were less likely to undergo coronary revascularization procedures than those without mental disorders [178]. 

### 4.13. Evidence for the Link between Poor Adherence Behaviors and CHD

Poor adherence to behaviors recommended for managing medical illnesses is well established as an important factor in determining medical outcomes for a range of diseases. For example, in a meta-analysis comparing high adherers with low adherers across a broad group of illnesses, treatment adherence accounted for 26% of the difference in outcomes [187]. Nonadherence to cardiovascular medications after ACS is clearly linked with poor medical outcomes. For example, Biondi-Zoccai et al. published a meta-analysis of the hazards of not adhering to aspirin among 50,279 CAD patients and showed that aspirin nonadherence was associated with a 3-fold higher MACE risk [188]. Gehi et al. showed that self-reported nonadherence to medications independently predicted adverse cardiac events (hazard ratio 2.3, 95% CI 1.3–4.3) in a cohort of stable CAD patients followed for nearly four years [189]. Rasmussen et al. showed an advantage in long-term survival in response to improved cardiovascular drug adherence after MI in a population-based sample [176]. Discontinuation of aspirin, statins, and beta blockers was independently associated with higher mortality (HR 3.81; 95% CI 1.88–7.72) in a cohort of 1,521 post-MI patients when medication use was assessed by patient-report [175]. 

### 4.14. Evidence for the Link between Medication Adherence and Depression

Although there are a number of potential behavioral mechanisms linking depression and post-ACS outcomes, poor medication adherence represents the most promising and best-supported mechanism for explaining this association. For example, in a study of 940 outpatients with stable CHD, 14% of patients with major depression reported that they were not taking their medication as prescribed, compared to five percent of patients without depression [190]. In contrast, in this same population of patients with stable CHD, no association was found between inflammation markers, a potential biological mechanism linking depression with post-ACS outcomes, and the complex phenotype of depression [191]. While these studies strengthen the causal link between nonadherence to cardiovascular medications and outcomes in post-ACS patients, they assessed medication adherence by self-report or by administrative databases rather than by using electronic medication monitoring, which is the gold-standard for measuring adherence. Furthermore, these studies did not consider patients from across the full spectrum of ACS diagnoses.

Among post-ACS patients, we have reported that 42% of persistently depressed patients took their prescribed aspirin less than three-fourths of the time, while only 10.5% of nondepressed patients demonstrated this level of nonadherence (*P* < .001) [192]. In a separate analysis of this same group of patients using a cross-lagged path analytic model, we showed that improvements in depressive symptoms in the first month after the ACS were associated with improved aspirin adherence in the subsequent two months (standardized direct effect −0.32, *P* = .016) [183], further establishing the close relationship between depression and medication adherence. 

### 4.15. Behavioral Mechanism Summary

Clearly, more detailed information on the mechanisms linking depression to CHD outcomes is needed to better tailor interventions. Confirming that electronically-monitored adherence is a mediator of this relationship in depressed patients will provide the evidence needed to develop tailored interventions for this subgroup of depressed post-ACS patients. Furthermore, assessing whether other patient and systems/physician behavioral factors additionally mediate poor outcomes can further inform the development of tailored interventions, and our understanding of how depression confers post-ACS prognostic risk.

## 5. Screening for Depression Comorbid with Coronary Heart Disease

### 5.1. Depression in CHD Patients Should Be Screened for and Treated, according to Numerous Guidelines

**Figure 2:**
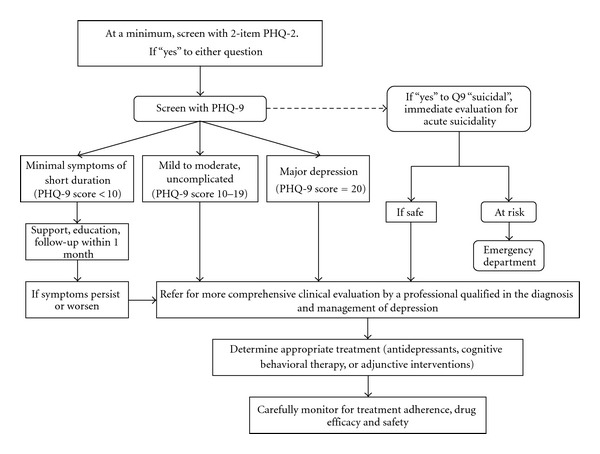
American Heart Association's Advisory for Depression Detection and Treatment (reprinted from Lichtman et al. [41]).

The strength of the depression to CHD outcome observational findings reviewed above has led many to advise routine depression screening for all CHD patients and referral for treatment when indicated, the approach incorporated into the recent advisory from the American Heart Association (AHA) [41] and endorsed by the American Psychiatric Association. Specifically, the advisory recommends administering a depression screening questionnaire to ACS patients and referring those who screen positive to a professional qualified to diagnose and manage depression (see Figure 2). We now have advisories/guidelines from AHA [41, 193], American Academy of Family Practitioners [194], European professional cardiology societies [195], and the British health care system [196] all endorsing depression screening in CHD patients, and then referral for treatment if depression is detected. However, there are no randomized controlled trials (RCTs) to inform this large, potentially expensive health care policy/guideline screening recommendation.

### 5.2. Perceived Barriers Interfere with Implementation of Depression Screening and Comprehensive Treatment in CHD Patients

In one recent examination of a quality initiative for depression screening, provider reported barriers to depression screening for CHD patients included: too little time, lack of professionals to refer to, and lack of education about logistics and usefulness [197]. Similarly, a 2012 education pretest offered by Medscape before a continuing medical education exercise on management of depression in patients with CHD revealed that the top three barriers reported by both cardiologists and primary care providers were: too little time, insufficient mental health network referrals, and lack of evidence to support this approach (January 2012; personal communication). 

### 5.3. Health-Related Quality of Life Improvements and Cost Effectiveness of Providing CHD Patients with the AHA Depression Screen and Treat Algorithm Are Unknown

There is a paucity of information from either observational or randomized controlled trials to estimate the costs and benefits of screening and treating depression in post-ACS patients. Only one randomized sertraline placebo-controlled trial, SADHART [198] (*n* = 369) has been used to estimate costs, but this did not include cost-effectiveness and *was not a depression screening RCT* [199]. This analysis noted that many costs were not represented, as they were not collected. 

A meta-analysis of RCTs testing depression screening with only notification of depression severity to primary care providers for primary care patients (NOT post-ACS patients) found that this strategy did not lead to increased detection or treatment of depression, and showed no impact on health-related quality of life, depressive symptoms, or other patient outcomes, including cost effectiveness [200]. An update of this Cochrane review found essentially the same disappointing results [201]. In a recent study examining the costs of conducting annual, lifetime, or every 5 year depression screening for primary care patients, the costs per QALY were unacceptable: the expected annual cost was $192,444 /QALY [202]. A nonstationary Markov model, using published literature, *found that no depression screening was preferable over annual depression screening*, and in the vast majority (99%) of scenarios, the cost per QALY was more than $50,000, with little expectation of patient benefit [202]. This heavy favoring of no screening makes sense in a primary care setting in which depression's association with clinical outcomes is less robust than in a post-ACS population. Thus, the evidence from RCTs in a primary care population have found that providing primary care patients with the depression screening and then treating have minimal quality of life improvements, and/or are cost ineffective. 

### 5.4. The Importance of RCTs in Evidence-Based Clinical Guidelines

Evidence-based practice guidelines are often distinguished from consensus-based practice guidelines or advisories, as the former systematically review all available research on the specified topic, and then grade the level of evidence to make a clinical recommendation [203, 204]. However, not all research designs are given equal weight; standards of evidence for guidelines have evolved to place greater emphasis on RCTs [205]. The reason for this is that these designs are the most replicable, have the fewest sources of bias, and all else being equal, have the greatest power to detect evidence that a screening practice or treatment results in a net benefit or net harm [206].

### 5.5. Screening Guidelines/Advisories in the Absence of RCT Evidence Have Recently Been Extensively Criticized (and Withdrawn)

As of the writing of this paper, a controversy is brewing over the reversal in one national screening guideline's recommendation [207–209]. Prostate cancer prevention screening had been considered equivocally beneficial by the U.S. Preventive Services Task Force (USPSTF) in 2002 [210] and 2008 [211], in large part because the observational data and common sense of this approach (the USPSTF found good evidence that PSA screening can detect early-stage prostate cancer). However, USPSTF on review of the most recent evidence, recently recommended *against* prostate-specific antigen-based screening for prostate cancer (grade D recommendation: there is moderate or high certainty that screening has no net benefit) for all men in the general US population, regardless of age [208]. They reversed their screening recommendation by reviewing the current body of evidence on the benefits and harms from recently conducted RCTs, data that were not available when screening was first thought to be useful. It is entirely possible that such a fate could await screening for depression in CHD patients. That is, current screening recommendations in clinical guidelines and AHA advisories are consensus-, rather than evidence-based, and the evidence, where it exists, does not favor depression screening [212]. 

## 6. Randomized Controlled Trials Treating Depression Comorbid with Coronary Heart Disease

The establishment of depression as a risk marker in patients with CHD prompted the National Heart, Lung, & Blood Institute to fund the Enhancing Recovery in CHD Patients (ENRICHD) trial, which randomized almost 2500 patients to determine whether treating depression and social isolation after acute MI improves event-free survival. There were no significant differences in all-cause mortality or nonfatal MI between the intervention and usual care arms of ENRICHD, nor in underpowered trials such as SADHART [198] and MIND-IT [213]. ENRICHD and these first generation phase II trials yielded only modest differences in depression between the treatment and control arms. Plausible reasons include: (1) the interventions were not efficacious, (2) the treatments were not well accepted, (3) the control conditions improved depression more than expected, and (4) the appropriate patient population was not studied. The ENRICHD investigators concluded that the next large, Phase III trial should be postponed until more efficacious depression treatments are available and the subtypes of depression that are most responsible for increased medical morbidity and mortality have been identified. Progress since then includes the STAR*D trial, [214] which demonstrated that aggressive, stepped-care delivery of existing therapies achieves better depression outcomes, several Phase II clinical trials that have demonstrated better depression outcomes in cardiac populations, and advances in our understanding of the characteristics of high-risk depression subtypes. 

Freedland et al. [215] conducted an RCT in 123 patients with major or minor depression who had recently undergone CABG surgery. The primary purpose of the trial was to determine the efficacy of 2 behavioral treatments (cognitive behavioral therapy [*CBT*] or supportive stress management) compared with usual care. The depression effect size for CBT at treatment end of 3 months was yielded a BDI depression effect size of **0.85 **(95% CI, 0.41–1.32). *This effect was maintained 6 months after the end of the trial*.

Huffman and others recently completed a randomized controlled trials of 175 hospitalized depressed cardiac patients [216]. They used a low-intensity depression collaborative care treatment, compared to usual care. The care was initiated in hospital, and then continued by telephone. Depression was assessed by the PHQ-9, using DSM-IV MDD criteria. All patients in the treatment arm received behavioral activation, and then by patient preference and/or prior treatment history received psychotherapy or pharmacotherapy. At 3 months (treatment end), treated patients had PHQ-9 score difference between group changes of **−3.43** (95% CI: −5.41–−1.45), and this was not quite maintained at 6 months (when no treatment was offered for the prior 3 months): **−1.77** (95% CI: −3.76–0.22). 

*The COPES II* [217, 218] post-ACS trial tested the acceptability and efficacy of 6-month *Stepped Depression Care*, a patient preference-driven, stepped algorithm depression intervention, to *Referred Depression Care*, in which depression screening was followed by physician notification of depression and encouragement to initiate depression treatment. The protocol included 157 CHD patients with depressive symptoms, and was not limited to those who met DSM-IV criteria for major depressive disorder. This strategy targeted the ACS patients who are at greatest mortality risk based on recent studies of depression and cardiac outcomes, namely those with postdischarge depressive symptoms. COPES II employed an aggressive, stepped care, patient preference, symptom-driven approach that increased the acceptability and efficacy of the depression intervention. Depression treatment acceptability was three times higher in the stepped depression care group than in the referred care group. The differential change in depression between groups yielded an effect size of **0.59 **(95% CI, 0.18–1.00). 

Whether the COPES intervention can be delivered as intended in multiple clinical centers was then addressed by *CODIACS I*, a feasibility/vanguard study conducted at 5 U.S. sites [219]. In this vanguard, the COPES protocol has been streamlined, case-finding made more efficient, and treatment delivery centralized and conducted by a web-based interface and then by telephone. Leading experts have concluded that it is time to conduct the next Phase III depression trial in CHD patients [220–223], as to date, treating depression after ACS has *not* resulted in improved cardiovascular outcomes [213, 224], primarily because we do not have large RCTs that actually test this question. 

## 7. Summary

CHD patients are quite likely to have subsyndromal or syndromal depression, which is associated with compromised quality of life, increased health care and societal costs, increased recurrence of CHD, and shortened life. We only have one early, adequately powered trial to test if depression reduction improved morbidity and mortality, and it did not [224]. We have few randomized controlled trials on which to base treatment decisions, but recent ones suggest that patient preference, collaborative care, and cognitive behavioral therapy all have promise for decreasing depression rates and improving quality of life. Clinicians should only screen for depression in their patients with CHD if they have the resources and system to conduct a thorough psychiatric diagnostic workup, refer or support patients found to be at imminent harm, and provide the depression treatment options needed to manage this relapsing, remitting disorder. For health care providers who do not have such a depression management system in place, referrals to appropriate sources of depression care is a better way to proceed. However, it behooves all of us to remain vigilant to the cardiovascular risk factor management issues that are faced by a depressed patient. Increasing structure, support systems, and other facilitators of adherence are important in these patients, as adherence is often a behavioral challenge they cannot manage. Finally, in the absence of more definitive tests of the biological and behavioral mechanisms by which depression may confer CHD risk in CHD patients, and in the absence of definitive trials testing if treating depression reduces this risk, we remain unsure if depression is a potent treatment target, or epiphenomena.
